# Emerging Concepts in the Paramedicine Literature to Inform the Revision of a Pan-Canadian Competency Framework for Paramedics: A Restricted Review

**DOI:** 10.7759/cureus.32864

**Published:** 2022-12-23

**Authors:** Jennifer Bolster, Priya Pithia, Alan M Batt

**Affiliations:** 1 Paramedicine, Monash University, Melbourne, AUS; 2 Clinical Governance and Professional Practice, British Columbia Emergency Health Services, Vancouver, CAN; 3 Centre for Research and Innovation, Fanshawe College, London, CAN

**Keywords:** emerging concept, competency framework, competency, emergency medical services, paramedic

## Abstract

The National Occupational Competency Profile (NOCP)-the competency framework for paramedics in Canada-is presently undergoing revision. Since the NOCP was published in 2011, paramedic practice, healthcare, and society have changed dramatically. To inform the revision, we sought to identify emerging concepts in the literature that would inform the development of competencies for paramedics. We conducted a restricted literature review and content analysis of all published and grey literature pertaining to or informing Canadian paramedicine from 2011 to 2022. Three authors performed a title, abstract, and full-text review to identify and label concepts informed by existing findings. A total of 302 articles were categorized into 11 emerging concepts related to competencies: inclusion, diversity, equity, and accessibility (IDEA) in paramedicine; social responsiveness, justice, equity, and access; anti-racism; healthy professionals; evidence-informed practice and systems; complex adaptive systems; learning environment; virtual care; clinical reasoning; adaptive expertise; and planetary health. This review identified emerging concepts to inform the development of the 2023 National Occupational Standard for Paramedics (NOSP). These concepts will inform data analysis, the development of group discussions, and competency identification.

## Introduction and background

The Paramedic Association of Canada (PAC) published the first National Occupational Competency Profile (NOCP) for paramedics in 2001 [1]. The NOCP has since been used by regulatory bodies, paramedic services, educators, and education accreditation agencies. Recognizing the shifting role of paramedicine in Canada in public safety and healthcare contexts, PAC renewed the NOCP in 2011 [1]. In 2016, additional work commissioned by PAC examined the roles paramedics should embody as part of their work (e.g., clinician, reflective practitioner) [2,3]. Given the central role that the NOCP plays within paramedicine in Canada, the planned 2023 revision must respond to evolving patient and societal needs through the identification of new competencies and the revision or removal of outdated competencies [4]. To this effect, in 2021, PAC partnered with the Canadian Standards Association (CSA) Group to manage the renewal of the NOCP and incorporate it into a new standard following the accredited processes of the Standards Council of Canada-the National Occupational Standard for Paramedics (NOSP).

The NOSP, while developed for the Canadian context, represents one of several ongoing international efforts to better understand and more accurately reflect contemporary paramedicine and paramedic practice [5-8]. We continue to experience a disconnect between paramedic practice and activities such as education, warranting a re-examination. For example, paramedics in Canada care for patients from differing environmental, social, and cultural contexts daily that are not sufficiently represented in existing practice documents [9]. Further, the perspectives and considerations of other minority and vulnerable populations that paramedics regularly care for have also historically been ignored. Examining and understanding contemporary (and future) paramedic practice in Canada will ensure that activities such as initial and continuing education, regulation, and assessment are better informed [10].

The development of the NOSP is guided by recent advances in the competency framework literature [4,9]. By adopting a systems-thinking approach, we recognize that we need to understand not only contemporary issues in paramedicine but also historical developments since 2011 and predicted future requirements to develop the NOSP. As such, this review aims to identify the emerging concepts in the paramedicine literature since 2011 to inform the development of the NOSP.

## Review

Methods

The purpose of this review is to provide new insights rather than summarize past research [11]. Therefore, we elected to perform a restricted review [12,13] focused on literature informing paramedicine in Canada, published from 2011 through 2022. A restricted review (an approach in which systematic review methods are streamlined and accelerated) [13] was considered appropriate due to the need for timely information and the aim of the review. Restricted review recommendations in this study included date restrictions, setting restrictions, database limits, single reviewer extraction, and narrative synthesis [13]. This review is not intended to be a comprehensive review of the literature. To begin, we defined emerging concepts for the purpose of this study as concepts that have emerged in the paramedicine literature since the 2011 NOCP but were not well represented within that document.

Next, we searched the Cumulated Index to Nursing and Allied Health Literature (CINAHL), Medical Literature Analysis and Retrieval System Online (MEDLINE), and Excerpta Medica Database (EMBASE) from 2011 to 2022. In addition, we conducted grey literature searches with guidance from the Canadian Agency for Drugs and Technologies in Health (CADTH) Grey Matters toolkit [14] on multiple organizational websites, Google Scholar [15], and Google Web. The search terms used included terms to describe paramedicine and paramedic service delivery (e.g., paramedic, EMT, EMS) [16], and an additional search was conducted with terms used to describe the Canadian context (e.g., Canada, Canadian). Subject headings were used where appropriate, and keywords and subject headings were adapted as required for individual databases. To complement these searches, we conducted a manual search and review of all 'Canadian Paramedicine' magazine issues from 2014 to 2022. This publication represents a central venue where paramedic discourse in Canada has occurred over the past four decades. We included articles of all types that discussed paramedicine in Canada but excluded news reports, media updates, those about a specific non-Canadian context, and conference abstracts. Studies in both English and French were included. Two reviewers (PP and JB) screened articles at the abstract and full-text levels, and a third reviewer (AB) resolved any conflicts. One reviewer (PP or JB) extracted the data, and a third reviewer (AB) performed a quality check on 20% of the articles. Records were imported into EndNote X20 (Clarivate, Philadelphia, PA, USA) for full-text retrieval, Covidence (Veritas Health Innovation, Melbourne, Australia) for screening and extraction, and Microsoft Excel 365 (Microsoft Corp., Redmond, WA, USA) for further analysis.

Analysis

We conducted continuous content analysis to identify emerging concepts that were related to or could inform paramedic competencies. Informed by the frameworks of Thoma et al. [17] and Van Melle [18], we modified their original emerging concepts to reflect: a) the differences in language between physicians and paramedics; and b) the methodology of the NOSP development [5,9]. For example, we changed 'physician humanism' [17] to 'healthy professionals' [5]. One author (AB) analyzed the extracted data and deductively categorized the results into emerging concepts. This was checked with the remaining authors, and we made minor amendments to the wording of concepts, but none were removed or amalgamated.

Positionality

Author JB is a paramedic researcher and faculty member whose clinical portfolio involves substance use and mental health. Author PP is a paramedic and research assistant. Author AB is a Ph.D. paramedic researcher and faculty member who teaches professional and emerging issues in paramedicine to undergraduate and graduate students.

Results

**Figure 1:**
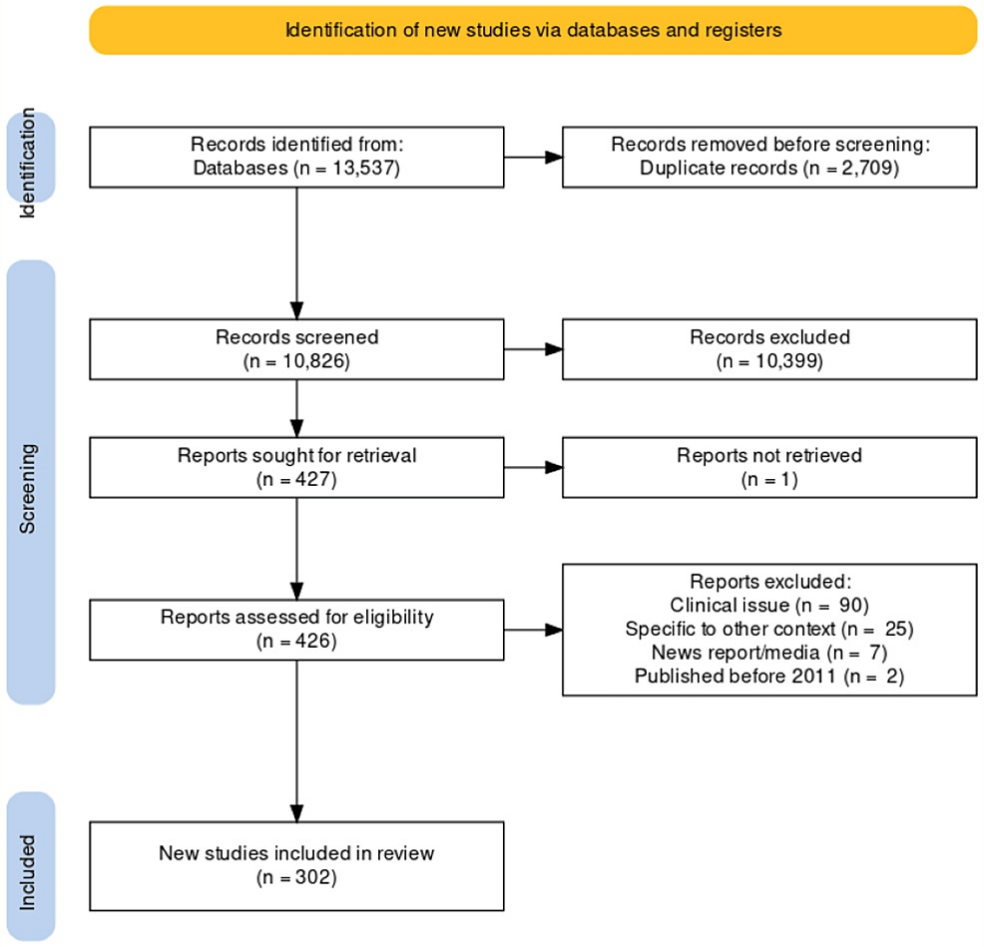
PRISMA flow diagram PRISMA: Preferred Reporting Items for Systematic Reviews and Meta-Analyses

The search strategy identified 13,537 studies. After the removal of duplicates, and title and abstract screening, we excluded 10,399 studies. The remaining 427 studies underwent full-text review, during which we excluded a further 117. A final total of 302 studies and reports (216 peer-reviewed articles; 86 grey literature items), informed this manuscript (see Figure 1). Articles were published from January 2011 to April 2022. A list of included studies can be accessed online at https://bit.ly/3FFXycZ [19].

Table 1 outlines the emerging concepts and the number of studies that were categorized by main concept and additional concept.

**Table 1:** Counts of emerging concepts in the literature

Emerging concept	Number of articles exploring a concept as the main concept (n=)	Number of articles exploring a concept as an additional concept (n=)
Inclusion, Diversity, Equity, and Accessibility (IDEA) in paramedicine	11	-
Social responsiveness, justice, equity, and access	49	15
Anti-racism	1	-
Healthy Professionals	60	2
Evidence-Informed Practice and Systems	76	10
Complex Adaptive Systems	25	23
Learning Environment	27	5
Virtual Care	2	-
Clinical Reasoning	26	9
Adaptive Expertise	21	5
Planetary Health	4	2
Total	302	71

Table 2 outlines the 11 emerging concepts, along with a description of the potential competencies discussed within each theme. Changes made to the names and definitions of the emerging concepts detailed in the existing literature were approved by all authors, and are referenced where applicable.

**Table 2:** Emerging concepts to inform the development of competencies for paramedics * Denotes an emerging concept that was amended to reflect differences in language between physicians and paramedics, or to reflect elements guiding the methodology of the NOSP development process. NOSP: National Occupational Standard for Paramedics

Emerging concept	Included topics	Principles and enabling factors	Description to inform the development of competencies
Inclusion, Diversity, Equity, and Accessibility (IDEA) in paramedicine*	Inclusion, diversity, equity; accessibility and disability; gender	Social responsiveness, shift in professional culture and identity, evidence-informed practice & systems	Competencies related to equity, diversity, inclusion, and accessibility within the paramedic population
Social responsiveness, justice, equity, and access*	Social determinants of health; equity of access to care; urban/rural disparity; structural competency	Health care along a health and social continuum, social responsiveness	Competencies related to access, equity, inclusion, and social justice within the care provided to patients [17]
Anti-racism	Anti-racism	Social responsiveness, health care along a health and social continuum	Competencies related to recognizing the existence of racism and actively seeking to identify, prevent, reduce, and remove the racially inequitable outcomes and power imbalances between groups and the structures that sustain these inequities [17]
Healthy Professionals*	Physical health; fitness; physical demands; mental health; empathy; quality of life	Healthy professionals	Competencies related to the experience of being a paramedic in a holistic sense incorporating physical health, mental health, wellbeing, spirituality, social and systemic supports.
Evidence-Informed Practice and Systems*	Big data; data informing practice; machine learning; technological advances; dispatch systems; evidence use	Integrate data environments, leverage advancing technology, intelligent access to and distribution of services, evidence-informed practice & systems	Competencies related to the role, collection, analysis and use of evidence and information in educational, service design and delivery, and clinical work.
Complex Adaptive Systems	Leadership; change; health systems science; community paramedicine program design; quality improvement; integrated care	Shift in professional culture and identity, integrated health care framework	Competencies related to the navigation of complexity within patient care, health and social care systems, and the integration of paramedic care.
Learning Environment*	Learning environment; research capacity; research priorities; culture; hidden curriculum; assessment	Enhance knowledge, quality based framework, continuous learning environment	Competencies related to clinical and non-clinical learning environments, and research to guide the profession.
Virtual Care	Telehealth; virtual clinical assessment	Evidence-informed practice & systems, intelligent access to and distribution of services	Competencies related to assessing and providing patient care in virtual environments.
Clinical Reasoning	Medical errors; patient safety; values-based approaches; patient/person-oriented care; efficiency; ethics; patient assessment	Patients & their communities first, quality-based framework	Competencies related to how paramedics think and function effectively in providing patient care.
Adaptive Expertise	Adaptive expertise; scene management; mentorship and sponsorship; team collaboration; reflective practice; self-regulation; professional development; autonomy	Promote shared understanding of paramedicine advance policy, regulation, legislation professional autonomy	Competencies related to the evolution, refinement, and development of the tools and skills required to practice effectively in a rapidly changing world. [17]
Planetary Health	Climate change; sustainability	Social responsiveness	Competencies related to the impact of climate and the environment on patients and paramedic services; and of patient care and paramedic service operations on climate and the environment.

Discussion

We sought to identify the emerging concepts in the paramedicine literature in Canada since 2011 to inform a revision and update of a pan-Canadian paramedic competency framework. To do this, we performed a restricted review and a content analysis informed by the existing literature. Our study identified 11 emerging concepts in the literature that could guide or inform the development of paramedic competencies. Each of these concepts is broad and similar to the findings of Thoma et al. [17]; they mirror broader influences in paramedicine, healthcare, and society as a whole over the last decade. We will now briefly discuss a number of observations in our findings.

Despite the impact and arguable importance of contemporary social issues, we observed a lack of literature related to a number of emerging concepts in paramedicine, in particular structural competency [20,21]. For example, despite the calls to action related to Indigenous healthcare in the final report of the Truth and Reconciliation Commission of Canada [22], only a handful of papers have explored the provision of paramedic care to Indigenous communities [23,24]. Yet Indigenous communities in Canada have faced health inequity and inequalities for decades [25,26]. Closely related to this, an area that received considerably less attention was the concept of anti-racism, with only one paper [27] outlining a call to action on anti-racism in paramedicine education and service delivery. We observed a similar lack of guiding literature related to the unique health and social care needs of members of the lesbian, gay, bisexual, and transgender (LGBTQ+) community, refugees, individuals experiencing homelessness, and many other marginalized and vulnerable populations [28-31]. Of particular concern was an observable lack of meaningful engagement with patients and caregivers from these communities in the literature we reviewed, suggesting that the needs and expectations of communities may be poorly understood. Therefore, the NOSP development process will not only need to identify the competencies required of paramedics when caring for members of diverse communities but will also need to have a concerted effort to engage, collaborate, and consult with such communities [20].

Climate change and sustainability in paramedicine have received little attention in the literature despite the serious consequences they hold for us all paramedics and communities alike. Medical education literature has acknowledged the need for current and future physicians to understand the impacts of climate change on health and wellness [32,33], and we suggest it is now past time for paramedicine in Canada to play a responsible role in this issue. While healthcare system and policy change lie outside the remit of developing the NOSP (e.g., paramedic services switching to hybrid or electric vehicles), we should endeavor to identify the competencies paramedics will need to respond to climate-related illnesses and injuries, which are predicted to increase in the coming years.

While the ongoing COVID-19 pandemic has increased the use of virtual care and telehealth delivery by paramedic services [34,35], we still lack a clear understanding of the role of paramedics as virtual care providers, primary care extenders, and facilitators of virtual care visits. The COVID-19 pandemic further emphasized the benefits of keeping people out of hospitals if they can be cared for at home or in their community [34]. We, therefore, need to better understand the competencies that paramedics require to work in this novel context of virtual care, as it is closely linked to the concepts of social justice and improving access to and equity of care among isolated and remote communities. Depending on the model of virtual care employed, paramedics may need to have additional or expanded competencies in assessment, communication, care planning, and multidisciplinary collaboration, as well as technological capabilities.

Finally, we observed an encouraging and considerable increase in literature exploring health and well-being in paramedicine over the past decade. We identified a total of 60 reports exploring paramedic physical health [36,37], safety [38,39], mental health [40,41], violence against paramedics [42], mental health supports and interventions [43], and family and social supports [44]. However, we observed a continued lack of understanding related to spirituality, holistic self-care, empathy, compassion, and the impacts and influences on paramedicine culture. The existing lack of competencies related to holistic self-care and wellness in the 2011 NOCP is a priority we aim to address when developing the NOSP.

Strengths and limitations

The strengths of this study rest in the transparent methods outlined in this manuscript, the guidance provided by existing publications [17,18], and the inclusion of grey literature representing paramedic community discourse over the last decade. We also used elements of the NOSP development process [2,4,5,9] to sensitize us and decrease the chance that important concepts were missed. However, this study has several limitations that we must acknowledge. First, we performed a restricted review that may not have captured all literature related to paramedicine in Canada. Despite the potential to have missed some themes, it is reassuring that no additional concepts were identified during analysis when compared to similar publications. Second, this study was conducted in parallel with a larger project related to standard development, and this placed logistical boundaries on the work (e.g., timeframes). Despite this, we suggest this study offers a comprehensive overview of emerging concepts that should be considered when developing the NOSP.

## Conclusions

This study identified 11 emerging concepts that should be considered when developing competencies as part of the NOSP: inclusion, diversity, equity, and accessibility (IDEA) in paramedicine; social responsiveness, justice, equity, and access; anti-racism; healthy professionals; evidence-informed practice and systems; complex adaptive systems; learning environment; virtual care; clinical reasoning; adaptive expertise; and planetary health. We hope that in addition to informing the NOSP, the publication of this work will create greater transparency around the development process. These emerging concepts would also benefit from further engagement among the paramedic community. In doing so, we may begin to understand how they intersect with our established understanding of the system of paramedicine.
